# New distribution records of subterranean crustaceans from cenotes in Yucatan (Mexico)

**DOI:** 10.3897/zookeys.911.47694

**Published:** 2020-02-12

**Authors:** Dorottya Angyal, Efraín M. Chávez-Solís, Luis A. Liévano-Beltrán, Benjamín Magaña, Nuno Simoes, Maite Mascaró

**Affiliations:** 1 Unidad Multidisciplinaria de Docencia e Investigación, Facultad de Ciencias, Universidad Nacional Autónoma de México, Puerto de abrigo S/N, C.P. 97356, Sisal, Yucatan, Mexico; 2 Department of Zoology, Hungarian Natural History Museum, Baross u. 13, 1088 Budapest, Hungary; 3 Posgrado en Ciencias Biológicas, Universidad Nacional Autónoma de México, Avenida Universidad 3000, Copilco-Universidad, Ciudad de México 04510, México; 4 Posgrado en Ciencias del Mar y Limnología, Universidad Nacional Autónoma de México, Avenida Universidad 3000, Copilco-Universidad, Ciudad de México 04510, México; 5 Laboratorio Nacional de Resiliencia Costera, Laboratorios Nacionales (LANRESC), CONACYT, Puerto de abrigo S/N, C.P. 97356, Sisal, Yucatan, Mexico; 6 International Chair for Ocean and Coastal Studies, Harte Research Institute, Texas A&M at Corpus Christi, Texas, USA

**Keywords:** anchialine ecosystems, barcode sequences, biodiversity, endemic, Eucarida, Peracarida, stygobiont, Yucatan Peninsula

## Abstract

New records of 14 stygobiont crustacean species pertaining to six Malacostraca orders from 32 cenotes are presented, with their associated caves of the state of Yucatan, Mexico, together with an individual account for each species. Species composition of most of the investigated cenotes is examined for the first time. A thermosbaenacean and two amphipod species were not formally recorded to the cenote ecosystems of the state of Yucatan prior to our research. Distribution data of a cirolanid isopod previously known only from its type locality is also provided. Barcodes of mitochondrial cytochrome c oxidase subunit I for the reported peracarid species previously lacking this information have been included in present study as tools for species identification and a baseline of further molecular genetic analyses.

## Introduction

’Cenotes’ (the local name for water-filled sinkholes) are typical karst features of the Yucatan Peninsula in Mexico. In many cases, far-reaching networks of submerged subterranean cave passages extend from them (Mercado-Salas et al. 2013). Due to the mixing of fresh and saline water, a distinct stratification can be observed inside these anchialine systems (Bishop et al. 2015). Intrusion of saline water is found deeper as the distance from the coastline increases (Bauer-Gottwein et al. 2011). Therefore, most inland cenotes within the state of Yucatan are exclusively freshwater systems, except for a few rather deep ones with haloclines below 50 m in depth, and those located near the northern coastline of the Peninsula (Álvarez et al. 2005; Angyal et al. 2018).

Anchialine ecosystems in Yucatan contain a crustacean-dominated fauna that is adapted to hypogene conditions, such as the lack of sunlight and the low food resource availability (Mejía-Ortíz et al. 2013). Stygobiont species are restricted to aquatic subterranean habitats (Botosaneanu 1986), and often exhibit conspicuous morphological adaptations to hypogene life, known as troglomorphisms. Such adaptations include structural reductions (e.g., loss of visual organs and pigmentation) or extensions (e.g., lengthening of appendages and complexity of sense organs) (Mejía-Ortíz et al. 2006; González et al. 2018) and physiological modifications (e.g., reduced metabolic rates and starvation resistance) (Hervant et al. 1999, 2001; Bishop and Iliffe 2009). In 2016, prior to our systematic sampling, 47 stygobiotic crustacean species had been reported from anchialine ecosystems of the Mexican federal states of the Yucatan Peninsula, of which 22 were known from cenotes and submerged caves of the state of Yucatan (e.g., Holsinger 1977; Kallmeyer and Carpenter 1996; Álvarez et al. 2005; Suárez-Morales et al. 2006). Fourteen percent of these species belong to the subclass Copepoda (9 spp.), while the remainder belong to the orders Mysida (1 sp.), Stygiomysida (2 spp.), Amphipoda (1 sp.), Isopoda (5 spp.), and Decapoda (4 spp.).

According to the database of the Secretaría de Desarrollo Sustentable (SDS Yucatan), there are more than 3,000 registered cenotes and caves within this state. Current efforts are being directed to complete the descriptions of all registered cenotes, despite that only a small fraction of them have been biologically investigated to date. Ongoing research and explorations are necessary to describe the true biodiversity of these subterranean habitats, their geographical patterns, and changes in time. Thus, our aim was to improve our knowledge on the distribution and ecology of the stygobiotic crustacean fauna of the cenotes and their associated cave passages in the state of Yucatan. We aimed to provide data from cenotes that had never been investigated from a zoological point of view in order to extend the geographical range of crustacean species distribution and contribute to a precise biodiversity mapping of stygofauna in Yucatan. Additionally, we intended to collect samples for molecular and morphological studies so as to gain and make available to the public mitochondrial cytochrome c oxidase subunit I sequences (COI) of species that were lacking barcode information, setting the standard for studies and tools for species identification.

## Materials and methods

### Sampling sites and sampling

**Figure 1.**
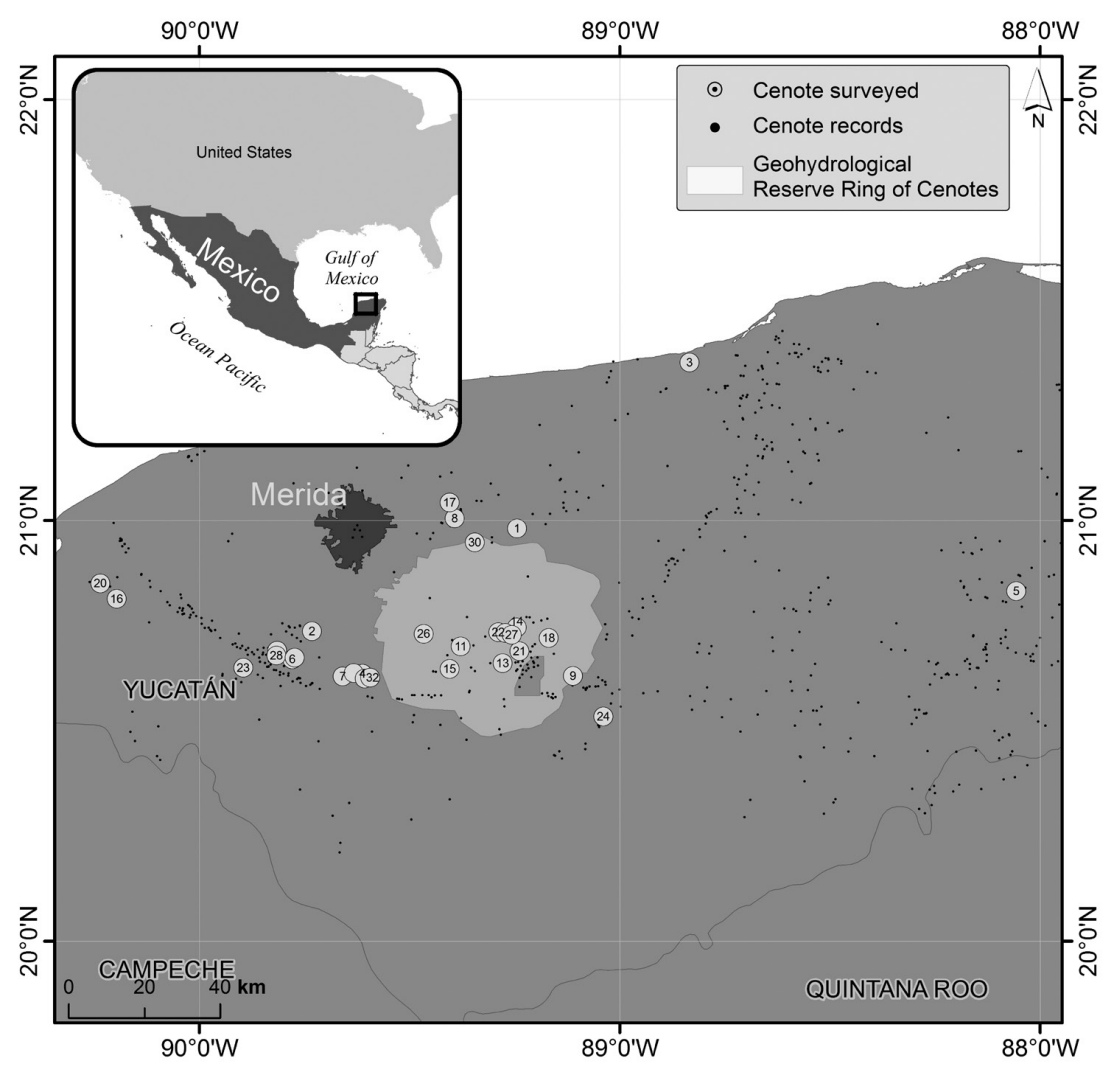
Map of the state of Yucatan and location of the 32 investigated cenotes. Details of the numbered cenotes can be found in Table 1. The light area represents the Geohydrological Reserve in Yucatan, while the dark area depicts the urban extension of the city of Merida.

We collected stygobiotic macro-crustaceans from 32 cenotes between May 2016 and January 2018 in cenotes of the state of Yucatan (shorter form: Yucatan) (Figure 1, Table 1). Most of the cenotes studied are several kilometers away from the coast and contain only freshwater. In contrast, some cenotes near the coast have a halocline that divides the cave into freshwater and saline water habitats. Some of the cenotes studied belong to the ’Ring of Cenotes’, a fracture zone with high density of sinkholes identified as the outer rim of the crater where the famous asteroid impacted Chicxulub 66 million years ago (González-Herrera et al. 2002; Bauer-Gottwein et al. 2011) (Figure 1). Macro-crustaceans were collected during scientific cave dives using 50 ml sample tubes and 10 cm diameter hand nets. Habitat data (e.g., depth, temperature, collected in cavern or cave, position relative to halocline) at the collection site of each individual was recorded along with photographs and video-recordings of the observed crustaceans and their habitats. All crustaceans were individually placed into 70 or 96% ethanol containing tubes immediately after collection. All specimens were collected under the permits of the Secretaría de Medio Ambiente y Recursos Naturales (SEMARNAT/SPGA/DGVS/05263/14; SEMARNAT/SPGA/DGVS/02068/17). The collected material was deposited in the Yucatán Collección de Crustáceos, Unidad Multidisciplinaria de Docencia e Investigación, Universidad Nacional Autónoma de México in Sisal (**UNAM UMDI-Sisal**), the Collección Nacional de Crustáceos, Instituto de Biología, **UNAM** in Mexico City, or in the Collection of Crustaceans of the Hungarian Natural History Museum (**HNHM**), Budapest.

**Table 1.** Location data and identification codes of the studied cenotes.

**Cenote nr. (see Figure 1 map)**	**Cenote name**	**CenoteAndo cenote code**	**Municipality**	**Settlement**	**Coordinates latitude / longitude**
1	Ayun-Nah	01980007Y_	Cacalchen	Cacalchen	20°58'49.6"N, 89°14'39.4"W
2	Bebelchen	00028064YC	Uman	Sanahcat	20°44'11.4"N, 89°43'55.4"W
3	Cervera	00090028YC	Dzilam de Bravo	Yalsihom	21°22'29.5"N, 88°50'01.8"W
4	Chihuo Hol	00080001YC	Abala	Mucuyche	20°38'06.1"N, 89°36'42.3"W
5	Dzalbay	00585085YC	Temozon	Dzalbay	20°49'53.4"N, 88°03'23.0"W
6	Dzonbakal	00125101YC	Uman	San Antonio Mulix	20°40'11.4"N, 89°46'43.9"W
7	Dzonotila	00168001YC	Abala	Mucuyche	20°37'44.0"N, 89°39'33.0"W
8	Flor de Liz	-	Tixkokob	Tixkokob	21°00'16.0"N, 89°23'33.0"W
9	Ixim Ha	00164037YC	Tixkakal	Tixkakal	20°37'49.0"N, 89°06'40.0"W
10	Kakuel	00142001YC	Abala	Mucuyche	20°37'40.3"N, 89°34'26.8"W
11	Kampepen	00042076YC	Tecoh	Chinquila	20°42'00.8"N, 89°22'41.6"W
12	Kankirixche	00002001YC	Abala	Mucuyche	20°38'13.8"N, 89°37'58.8"W
13	Kankal	-	Homun	Homun	20°39'38.3"N, 89°16'42.5"W
14	Kanun	01730036Y_	Homun	Homun	20°44'44.2"N, 89°14'40.7"W
15	Nayah	00009076YC	Tecoh	Pixyah	20°38'47.5"N, 89°24'16.9"W
16	Noh’Chunck	00229011YC	Chunchumil	Celestun	20°48'48.5"N, 90°11'47.8"W
17	Nohmozon	00010076YC	Tecoh	Pixyah	20°62’32.5”N, 89°38’42.0”W
18	Pixton	00064064YC	Huhi	Huhi	20°43'13.3"N, 89°10'08.5"W
19	Pol Box	00321023YC	Chochola	Chochola	20°41'24.3"N, 89°48'54.5"W
20	Sabtun 1	00230011YC	Chunchumil	Celestun	20°51'00.7"N, 90°14'08.1"W
21	San Elias	01171036Y_	Homun	Homun	20°41'21.0"N, 89°14'19.0"W
22	San Juan	00063036YC	Homun	Homun	20°44'02.6"N, 89°17'18.6"W
23	Santito	00108045YC	Kopoma	Kopoma	20°38'58.1"N, 89°53'44.3"W
24	El Virgen	-	Sotuta	Sotuta	20°32'01.9"N, 89°02'19.4"W
25	Tres Oches	-	Homun	Homun	20°43'55.7"N, 89°16'20.0"W
26	Tza Itza	00050076YC	Tecoh	Tecoh	20°43'49.1"N, 89°27'57.9"W
27	Xaan	00423036YC	Homun	Homun	20°43'39.3"N, 89°15'24.6"W
28	X’baba	00162023YC	Chochola	Chochola	20°40'42.5"N, 89°49'00.7"W
29	X-Batun	00005023YC	Uman	San Antonio Mulix	20°40'23.8"N, 89°46'22.8"W
30	X’kokob	00650093YC	Ekmul	Ekmul	20°56'51.0"N, 89°20'41.0"W
31	Yaal Utsil	00003001YC	Abala	Mucuyche	20°37'26.0"N, 89°36'24.0"W
32	Yax-Kis	00091001YC	Abala	Mucuyche	20°37'33.7"N, 89°35'35.7"W

### Morphological analysis

Individuals were examined using a stereo-microscope. Specimens of thermosbaenaceans, stygiomysids, mysids, and amphipods were studied as follows: cleared and stained exoskeletons were dissected under a Leica M125 stereo microscope. The dissections were then mounted on slides and examined using a Leica DM 1000 compound light microscope (Fišer et al. 2009; Angyal et al. 2015). For the identification of the collected material the following publications were used: Álvarez et al. 2005; Álvarez and Iliffe 2008; Angyal et al. 2018; Botosaneanu and Iliffe 1999, 2000, 2002, 2006; Bowman 1966, 1977; Bruce 1986; Creaser 1936; Hobbs and Hobbs 1976; Hobbs et al. 1977; Hobbs 1979; Holsinger 1977, 1990; Horwitz et al. 1995; Kallmeyer and Carpenter 1996; Lowry and Myers 2013; Meland et al. 2015; Pérez-Aranda 1983a, 1983b, 1984a, 1984b; Tinnizi and Quddusi 1993; Wagner 1994. Photographs were made using an OMAX 14 OMP digital USB microscope camera, a Nikon D5300, and a Nikon D7000 with 60 mm macro lens.

### Molecular studies (COI barcode sequences)

DNA extraction of the peracarids studied was performed using QIAamp DNA Microkit (QIAGEN), following the manufacturer’s instructions. A few pereopods of each animal provided the necessary material to extract DNA. For PCR amplification of mitochondrial COI, we used the primer pair LCO 1490 and HCO 2198 (Folmer et al. 1994). PCR reactions (25 μl) contained 13.85 μl mQ water, 2.5 μl 10× PCR buffer, 2.5 μl dNTP mix (2mM), 1.5 μl of each primers (5μM), 0.15 μl Fermentas Dream Taq (5U/ μl), and 3 μl DNA extract. PCR temperature conditions were set as follows: initial denaturation for 3 min at 94 °C, denaturation for 45 sec at 94 °C, hybridization for 45 sec at 48 °C, and polymerization for 1 min at 72 °C. After thirty cycles, a final extension for 3 min at 72 °C was performed. PCR products were purified using Exo SAP-IT Express PCR Product Cleanup (Affymetrix) according to the manufacturer’s instructions. The fragments were sequenced in both directions using PCR amplification primers with an ABI 3130 sequencer. Contigs were assembled and sequences were edited using BioEdit 7.1.11 sequence alignment editor software (Hall 1999): chromatograms of complement reverse and forward strings were compared, gaps were eliminated, while indels and stop codons were checked. 605-651 bp COI barcode sequences have been uploaded to the NCBI GenBank database. Accession numbers and localities are listed in Table 2.

**Table 2.** Locality data and GenBank accession number of COI gene fragments of one individual of each newly collected stygobiotic peracarid species.

**Taxon**	**Locality (cenote)**	**Voucher**	**GenBank accession nr.**	**Cited in**
*Tulumella unidens* (Thermosbaenacea)	Sabtun 1	YUC-CC-255-11-004-656	MK 900685	present study
*Stygiomysis cokei* (Stygiomysida)	Dzonotila	YUC-CC-255-11-004-638	MK 900690	present study
Stygiomysis cf. holthuisi (Stygiomysida)	Kankal	YUC-CC-255-11-004-621	MK 900689	present study
*Antromysis cenotensis* (Mysida)	Pol Box	YUC-CC-255-11-004-694	MK981568	present study
*Mayaweckelia troglomorpha* (Amphipoda)	Dzonbakal	CNR 34392	MF589977	Angyal et al. 2018
*Mayaweckelia cenoticola* (Amphipoda)	Ayun-Nah	YUC-CC-255-11-003923	MF589975	Angyal et al. 2018
*Tuluweckelia cernua* (Amphipoda)	Kankirixche	YUC-CC-255-11-003924	MF589983	Angyal et al. 2018
*Creaseriella anops* (Isopoda)	Tza Itza	HNHM-YUC_Isopoda-01	MK 900687	present study
*Yucatalana robustispina* (Isopoda)	Kankirixche	YUC-CC-255-11-004-715	MK 900686	present study
*Cirolana yunca* (Isopoda)	Tres Oches	HNHM-YUC-Isopoda-02	MK 900688	present study

## Results

**Figure 2.**
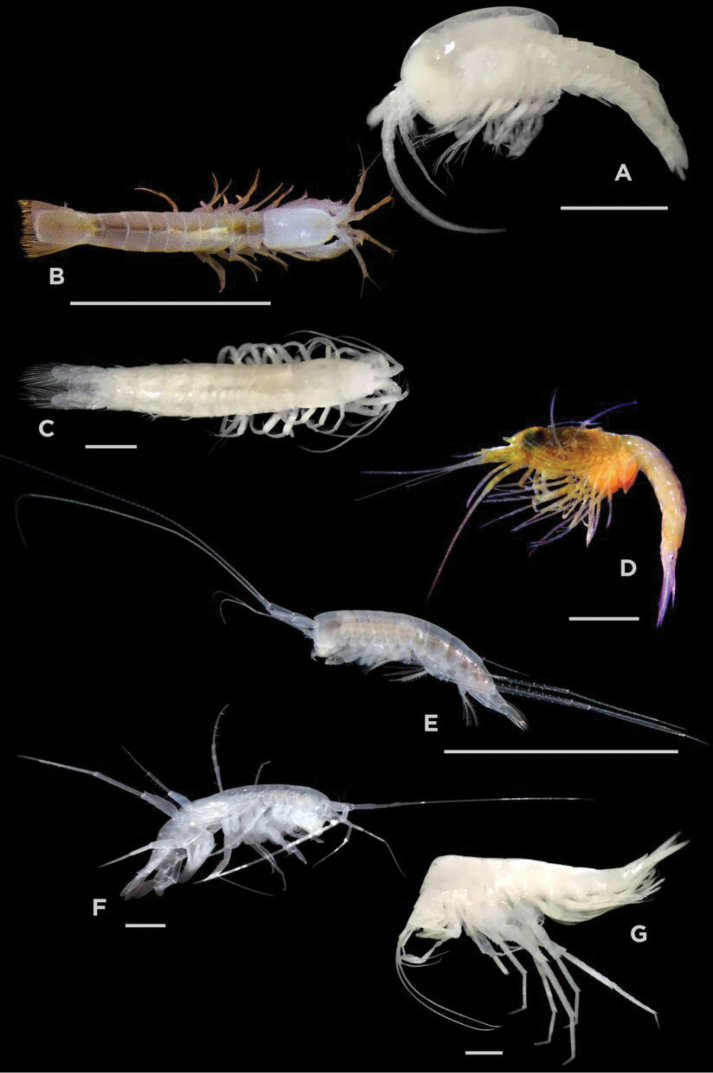
**A***Tulumella unidens* (Thermosbaenacea) **B***Stygiomysis cokei* (Stygiomysida) **C**Stygiomysis cf. holthuisi (Stygiomysida) **D***Antromysis cenotensis* (Mysida) **E***Mayaweckelia troglomorpha* (Amphipoda) **F***Mayaweckelia cenoticola* (Amphipoda) **G***Tuluweckelia cernua* (Amphipoda). Scale bars: 1 mm (**A, C, D, F, G)**; 10 mm **(B, E)**.

**Figure 3.**
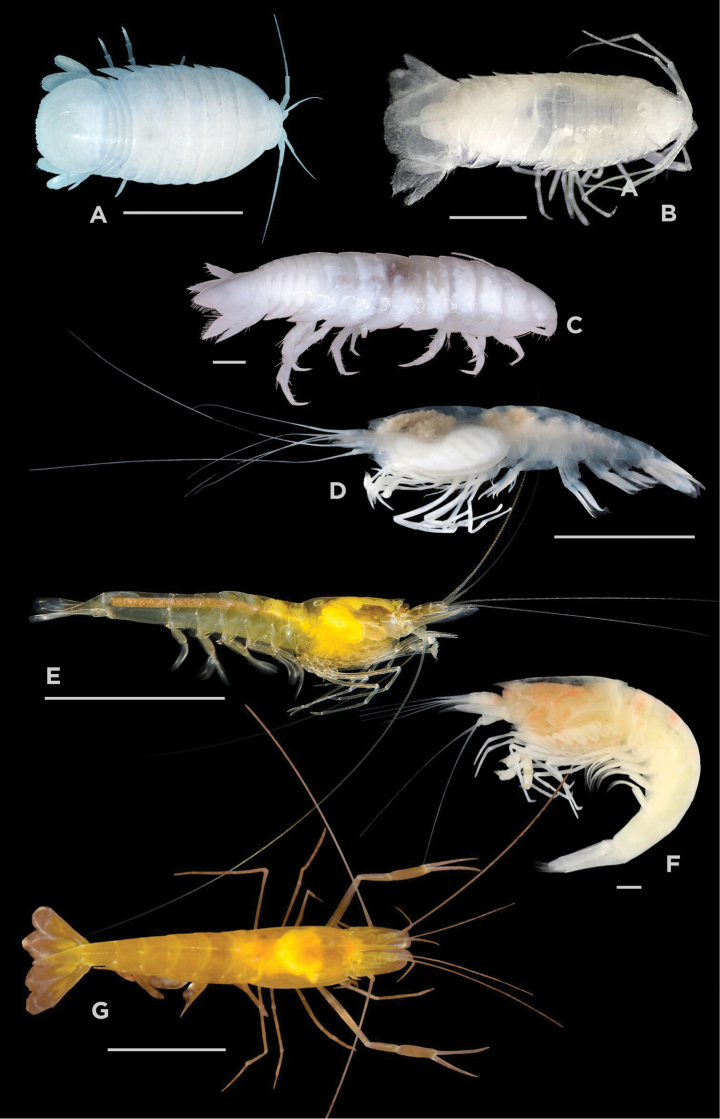
**A***Creaseriella anops* (Isopoda) **B***Yucatalana robustispina* (Isopoda); **C***Cirolana yunca* (Isopoda) **D***Typhlatya dzilamensis* (Decapoda) **E***Typhlatya mitchelli* (Decapoda) **F***Typhlatya pearsei* (Decapoda) **G***Creaseria morleyi* (Decapoda). Scale bars: 1 mm (**B, C, F**); 10 mm **(A, D, E, G**).

A total of 14 stygobiont crustacean species, belonging to six Malacostraca orders, was collected (Figures 2, 3). New records of each species at each cenote were assessed after an exhaustive literature investigation (Table 3). This evaluation was based only on the collected material that has been deposited in scientific collections. Additional data based on observations, however, are mentioned in the “Remarks” section in each case. An individual account for each species is subsequently discussed. 605-651 base-pair COI barcode sequences of the analyzed species (Table 2) were obtained and uploaded to NCBI GenBank (https://www.ncbi.nlm.nih.gov/genbank/).

**Table 3.** Records of stygobiotic crustacean species collected between May 2016 and January 2018 in 32 cenotes of Yucatan. Bold-faced locality names represent new records for the cenote, while bold-faced locality names with an asterisk (*) represent new records for the state of Yucatan.

**Taxon**	**Cenote**
** THERMOSBAENACEA **	
*Tulumella unidens* Bowman & Iliffe, 1988	Cervera*, Sabtun 1*
** STYGIOMYSIDA **	
*Stygiomysis cokei* Kallmeyer & Carpenther, 1996	**Tres Oches**, **San Elias**, Dzonotila, **Yax-Kis**
Stygiomysis cf. holthuisi (Gordon, 1958)	**Tres Oches**, **Tza Itza, X-Batun**, **Kanun**, **Kankirixche**, **Kakuel**, **Santito**, **Pol Box**, **Kankal**, **Flor de Liz**, **Bebelchen**, **Chihuo Hol**, **Yax Kis**
** MYSIDA **	
*Antromysis cenotensis* Creaser, 1936	**Tza Itza**, **Dzonbakal**, **Nayah**, **Kampepen**, **Kanun**, **Xaan**, **Kakuel**, Kankirixche, **Santito**, **Pol Box**, **Kankal**, **Dzonotila**, **Ixim Ha**, **Noh’Chunck**, **X’kokob**, **Flor de Liz**, **Pixton**, **Bebelchen**, **El Virgen**, **Chihuo Hol**
** AMPHIPODA **	
*Mayaweckelia cenoticola* Holsinger, 1977	**Ayun-Nah**, **Dzonotila**, **Ixim Ha**, **Bebelchen**
*Mayaweckelia troglomorpha* Angyal, 2018	Dzonbakal*, Kanun*, Xaan*, Kankirixche*, Dzonotila*, X’kokob*, Chihuo Hol*, Yax-Kis*
*Tuluweckelia cernua* Holsinger, 1990	San Juan*, Dzonbakal*, Tres Oches*, Xaan*, Kakuel*, Kankirixche*, Santito*, X’baba*, Sabtun 1*, Pixton*, Yax-Kis*
** ISOPODA **	
*Creaseriella anops* (Creaser, 1936)	**San Juan**, Cervera, **Tza Itza**, **Tres Oches**, Kankirixche, **Chihuo Hol**
*Yucatalana robustispina* Botosaneanu & Iliffe, 1999	**Xaan**, Kakuel, Kankirixche, **Yaal Utsil**, **Tza Itza, Pol Box**, **Dzonotila**, **X’baba**, **El Virgen**, **Chihuo Hol**, **Yax Kis**
*Cirolana yunca* (Botosaneanu & Iliffe, 2000)	**Tres Oches**, **X’baba**, **Chihuo Hol**
** DECAPODA **	
*Typhlatya dzilamensis* Alvarez, Iliffe & Villalobos, 2005	Cervera, **Sabtun 1**
*Typhlatya mitchelli* Hobbs & Hobbs, 1976	San Juan, Tza Itza, **Dzonbakal**, **Kampepen**, **Ayun-Nah**, **Tres Oches**, Kakuel, Kankirixche, **Sabtun 1**, **Bebelchen**, **El Virgen**, **Chihuo Hol**
*Typhlatya pearsei* Creaser, 1936	**Tres Oches**, **Xaan**, Kankirixche, Nohmozon
*Creaseria morleyi* (Creaser, 1936)	Tza Itza, **Kampepen**, **Kakuel**, Kankirixche, **Santito**, **Kankal**, **Bebelchen**, **El Virgen**, **Dzalbay**

### Subphylum: Crustacea

#### 

**Class: Malacostraca**

**Superorder: Peracarida**

**Order: Thermosbaenacea**

**Family: Tulumellidae**

##### Tulumella unidens

Taxon classificationAnimaliaThermosbaenaceaTulumellidae

Bowman & Iliffe, 1988

8D0EFF97-EF86-5F7B-A670-2A27CB46FFF5

Figure 2A

###### Material examined.

4 individuals; **Cenote Cervera**, depth 25.6-26.2 m, cave, in hydrogen sulfide layer, around and below halocline, 26 °C, Yalsihom, Yucatan, Mexico; 8 May 2016; colls. D. Angyal & E. Chávez Solís. 4 individuals; **Cenote Sabtun 1**, depth 24.0-25.0 m, cavern, above and around halocline, 25 °C, Chunchumil, Yucatan, Mexico; 10 December 2017; colls. D. Angyal, E. Chávez Solís, S. Drs, Q. Hernández & S. Reyes.

###### Previous distribution.

Iliffe 1992; Iliffe 1993; Bowman and Iliffe 1988; Rocha et al. 1998; Pohlman et al. 2000; Pesce and Iliffe 2002; Álvarez et al. 2015; Olesen et al 2015; Benítez et al. 2019.

Type locality is Cenote Naharon (Cristal) in Quintana Roo. This species had only been reported from Quintana Roo from cenotes Calavera (Temple of Doom), Mayan Blue, Actun Ha (Carwash), Muknal, Na’ach Wennen Ha, Bang, Odyssey, Tabano, and Quebrada.

###### Remarks.

Our findings extend the distribution area of this thermosbaenacean, previously endemic to Quintana Roo, to the cenotes located in the coastal areas north of Dzilam de Bravo and the east of Celestun. It is most likely that this species has a coastal distribution along the anchialine systems of the Yucatan Peninsula. Previous records were reported from cenotes located 2-10 km from the coastline near Tulum, where they occurred mostly above and at the halocline (Álvarez & Iliffe 2008; Álvarez et al. 2015; Benítez et al. 2019). In Cenote Cervera, 3.6 km inland from the northern coast of the Yucatan Peninsula, we observed individuals both above and below the halocline, as well as in the hydrogen sulfide layer.

#### 

**Order: Stygiomysida**

**Family: Stygiomysidae**

##### Stygiomysis cokei

Taxon classificationAnimaliaStygiomysidaStygiomysidae

Kallmeyer & Carpenther, 1996

E59BDF53-A0A5-5F28-94D1-E0BDA3BCA483

Figure 2B

###### Material examined.

1 individual; **Cenote Tres Oches**, depth 21.6 m, cave, freshwater, 27 °C, Homun, Yucatan, Mexico; 5 June 2016; colls. D. Angyal & E. Chávez Solís. 2 individuals, **Cenote San Elias**, depth 28.2 m and 32.0 m, cavern, freshwater, 26 °C, Homun, Yucatan, Mexico; 19 November 2017; colls. D. Angyal, E. Chávez Solís, S. Drs & L. Liévano. 2 individuals; **Dzonotila**, depth 20.8 m and 28.0 m, cavern, freshwater, 27 °C, Mucuyche, Yucatan, Mexico; 20 November 2017; colls. D. Angyal, E. Chávez Solís, S. Drs & B. Magaña. 1 individual; **Yax-Kis**, depth 12.1 m and 27.0 m, cave, freshwater, 27 °C, Mucuyche, Yucatan, Mexico; 27 January 2018; colls. D. Angyal, S. Drs & L. Liévano.

###### Previous distribution.

Kallmeyer and Carpenter 1996; Pesce and Iliffe 2002; Álvarez and Iliffe 2008; Álvarez et al. 2015; Benítez et al. 2019.

Type locality is Cenote Calavera (Temple of Doom) in Quintana Roo. Further known localities in Quintana Roo are cenotes Mayan Blue, Naharon (Cristal), Escondido, Actun Ha (Carwash), Actun Ko, Na’ach Wennen Ha, Muknal and Tabano. From Yucatan the species was known from cenotes Papakal, San Eduardo, Kankirixche, Yaal Utsil and Dzonotila.

###### Remarks.

Our records show that this species is distributed in cenotes of central Yucatan and along the Ring of Cenotes. Among the two *Stygiomysis* species of the region, *S. cokei* proved to be rarer than Stygiomysis cf. holthuisi. New occurrences were recoded between 12-32 m deep in freshwater. In cenotes San Elias, Dzonotila and Yax-Kis it co-occurred with S. cf. holthuisi. Previously the species had also been reported in brackish habitats (Álvarez and Iliffe 2008; Álvarez et al. 2015).

##### Stygiomysis cf. holthuisi

Taxon classificationAnimaliaStygiomysidaStygiomysidae

(Gordon, 1958)

E62795EA-C7C1-5D0C-8B95-C214F62869C0

Figure 2C

###### Material examined.

2 individuals; **Cenote Tres Oches**, depth 21.6 m, cave, freshwater, 27 °C, Homun, Yucatan, Mexico; 5 June 2016; colls. D. Angyal & E. Chávez Solís. 1 individual; **Cenote Tza Itza**, depth 18.9 m, cavern, freshwater, 27 °C, Tecoh, Yucatan, Mexico; 10 May 2016; colls. D. Angyal & E. Chávez Solís. 1 individual; **Cenote X-Batun**, depth 19.3 m, cavern, freshwater, 27 °C, San Antonio Mulix, Yucatan, Mexico; 14 May 2016; colls. R. Acosta, D. Angyal, J. Baduy & S. Reyes. 3 individuals; **Cenote Kanun**, depth 10.9-13.0 m, cave, freshwater, 26 °C, Homun, Yucatan, Mexico; 4 June 2016; colls. R. Acosta, D. Angyal, J. Baduy, B. Magaña & S. Reyes. 1 individual; **Cenote Kakuel**, depth 29.8 m, cave, freshwater, 27 °C, Mucuyche, Yucatan, Mexico; 10 June 2016; colls. D. Angyal & E. Chávez Solís. 1 individual; **Cenote Kankirixche**, depth 3 m, cavern, freshwater, 27 °C, Mucuyche, Yucatan, Mexico; 11 June 2016; colls. D. Angyal & E. Chávez Solís. 1 individual; **Cenote Santito**, depth 5.4 m, cavern, freshwater, 27 °C, Kopoma, Yucatan, Mexico; 10 November 2017; colls. D. Angyal, D. Drs & L. Liévano. 1 individual; **Cenote Pol Box**, depth 3.0 m, cavern, freshwater, 27 °C, Chochola, Yucatan, Mexico; 12 November 2017; colls. D. Angyal, S. Drs, L. Liévano & E. Sosa. 4 individuals; **Cenote Kankal**, depth 6.0-27.0 m, cavern, freshwater, 25 °C, Homun, Yucatan, Mexico; 12 November 2017; colls. D. Angyal, S. Drs, L. Liévano & E. Sosa. 2 individuals; **Cenote Flor de Liz**, depth 3.0 m, cavern, freshwater, 27 °C, Tixkokob, Yucatan, Mexico; 17 December 2017; colls. D. Angyal, S. Drs, L. Liévano & S. Reyes. 1 individual; **Cenote Bebelchen**, depth 30.0 m, cavern, freshwater, 25 °C, Sanahcat, Yucatan, Mexico; 18 December 2017; colls. D. Angyal, S. Drs, L. Liévano & S. Reyes. 2 individuals; **Cenote Chihuo Hol**, depth 16.0 and 25.0 m, cavern, freshwater, 27 °C, Mucuyche, Yucatan, Mexico; 26 January 2018; colls. D. Angyal, S. Drs, L. Liévano, B. Magaña & N. Simoes. 3 individuals; **Yax Kis**, depth 9.0-25.0 m, cave, freshwater, 27 °C, Mucuyche, Yucatan, Mexico; 27 January 2018; colls. D. Angyal, S. Drs & L. Liévano.

###### Previous distribution.

Gordon 1958; Botosaneanu 1980; Bowman et al. 1984; Pesce and Iliffe 2002; Álvarez and Iliffe 2008, Álvarez et al. 2015, Benítez et al. 2019.

Type locality is Devil's Hole, St. Martin, Lesser Antilles (France). The species is known from the Bahamas, Anguilla, Puerto Rico, and the Yucatan Peninsula. In Quintana Roo S. cf. holthuisi was recorded from cenotes Mayan Blue, Casa Cenote, Na’ach Wennen Ha, Bang, Odyssey, Muknal, and Tabano. From Yucatan the species was previously known only from a single locality, Cenote Mucuyche.

###### Remarks.

We have also recorded the species from cenotes Yaal Utsil, San Elias, and Dzonotila in freshwater bodies in both cavern and cave sections, between 3 and 30 m deep. Álvarez and Iliffe (2008) and Álvarez et al. (2015) reported observations in both freshwater and around the halocline from cenotes in Quintana Roo.

#### 

**Order: Mysida**

**Family: Mysidae**

##### Antromysis cenotensis

Taxon classificationAnimaliaMysidaMysidae

Creaser, 1936

500981D5-83C4-596F-8942-4873DE75F275

Figure 2D

###### Material examined.

21 individuals; **Cenote Tza Itza**, depth 12.7-13.5 m, cavern, freshwater, 27 °C, Tecoh, Yucatan, Mexico; 10 May 2016; colls. D. Angyal & E. Chávez Solís. 1 individual; **Cenote Dzonbakal**, depth 25.3 m, cavern, freshwater, 27 °C, San Antonio Mulix, Yucatan, Mexico; 14 May 2016; colls. R. Acosta, D. Angyal, J. Baduy & S. Reyes. 1 individual; **Cenote Nayah**, depth 27.9 m, entrance of cave part, freshwater, 26 °C, Pixyah, Yucatan, Mexico; 17 May 2016; colls. D. Angyal & B. Magaña. 3 individuals; **Cenote Kampepen**, depth 9.3-12.5 m, cavern, freshwater, 27 °C, Chinquila, Yucatan, Mexico; 17 May 2016; colls. D. Angyal & B. Magaña. 4 individuals; **Cenote Kanun**, depth 0.5 m, cenote entrance, freshwater, 26 °C, Homun, Yucatan, Mexico; 4 June 2016; colls. R. Acosta, D. Angyal, J. Baduy, B. Magaña & S. Reyes. 4 individuals; **Cenote Xaan**, depth 22.2-24.2 m, cavern and cave, freshwater, 27 °C, Homun, Yucatan, Mexico; 9 June 2016; colls. D. Angyal & E. Chávez Solís. 15 individuals; **Cenote Kakuel**, depth 7.2-10.8 m, cavern, freshwater, 27 °C, Homun, Yucatan, Mexico; 10 June 2016; colls. D. Angyal & E. Chávez Solís. 1 individual; **Cenote Kankirixche**, depth 9.0 m, cavern, freshwater, 27 °C, Mucuyche, Yucatan, Mexico; 11 June 2016; colls. D. Angyal & E. Chávez Solís. 4 individuals; **Cenote Kankirixche**, depth 10.0-25.0 m, cavern, freshwater, 27 °C, Mucuyche, Yucatan, Mexico; 25 January 2018; colls. D. Angyal, S. Drs, B. Magaña & L. Liévano. 18 individuals; **Cenote Santito**, depth 0.2-1.0 m, cavern, freshwater, 27 °C, Kopoma, Yucatan, Mexico; 10 November 2017; colls. D. Angyal, S. Drs & L. Liévano. 17 individuals; **Cenote Pol Box**, depth 5.2-9.3 m, cavern, freshwater, 27 °C, Chochola, Yucatan, Mexico; 12 November 2017; colls. D. Angyal, S. Drs, L. Liévano & E. Sosa. 1 individual; **Cenote Kankal**, depth 24.6 m, cavern, freshwater, 25 °C, Homun, Yucatan, Mexico; 18 November 2017; colls. D. Angyal, E. Chávez Solís, S. Drs & L. Liévano. 21 individuals; **Dzonotila**, depth 3.0-27.0 m, cavern, freshwater, 27 °C, Mucuyche, Yucatan, Mexico; 20 November 2017; colls. D. Angyal, E. Chávez Solís, S. Drs & B. Magaña. 5 individuals; **Cenote Ixim Ha**, depth 10.0 m, cavern, freshwater, 25 °C, Tixkakal, Yucatan, Mexico; 25 November 2017; colls. D. Angyal, E. Chávez Solís, S. Drs, L. Liévano & E. Sosa. 1 individual; **Cenote Noh’Chunck**, depth 12.0 m, cavern, freshwater, 25 °C, Chunchumil, Yucatan, Mexico; 25 November 2017; colls. D. Angyal, E. Chávez Solís, S. Drs, Q. Hernández & S. Reyes. 11 individuals; **Cenote X’kokob**, depth 1.0-4.0 m, cavern, freshwater, 25 °C, Ekmul, Yucatan, Mexico; 17 December 2017; colls. D. Angyal, S. Drs, L. Liévano & S. Reyes. 14 individuals; **Cenote Flor de Liz**, depth 0.3-3.0 m, cavern, freshwater, 27 °C, Tixkokob, Yucatan, Mexico; 17 December 2017; colls. D. Angyal, S. Drs, L. Liévano & S. Reyes. 19 individuals; **Cenote Pixton**, depth 3.0 m, cavern, freshwater, 27 °C, Huhi, Yucatan, Mexico; 18 December 2017; colls. D. Angyal & L. Liévano. 11 individuals; **Cenote Bebelchen**, depth 27.0 m, cavern, freshwater, 25 °C, Sanahcat, Yucatan, Mexico; 18 December 2017; colls. D. Angyal, L. Liévano & S. Reyes. 6 individuals; **Cenote El Virgen**, depth 25.0 m, cavern, freshwater, 26 °C, Sotuta, Yucatan, Mexico; 20 December 2017; colls. L. Liévano & N. Simoes. 3 individuals; **Cenote Chihuo Hol**, depth 11.0 m, cavern, freshwater, 27 °C, Mucuyche, Yucatan, Mexico; 20 December 2017; colls. D. Angyal, S. Drs, B. Magaña, L. Liévano & N. Simoes.

###### Previous distribution.

Ceaser 1936, 1938; Nicholas 1962; Bowman 1977; Redell 1977, 1981; Holsinger 1990; Iliffe 1992, 1993; Fiers et al. 1996; Rocha et al. 1998, 2000; Suárez-Morales and Rivera 1998; Pohlman et al. 2000; Pesce and Iliffe 2002; Schmitter-Soto et al. 2002; Álvarez and Iliffe 2008; Álvarez et al. 2015; Benítez et al. 2019.

Type locality is Grutas de Balankanche (Yucatan). Widely distributed in the central and northern parts of the Yucatan Peninsula, known from several wells, cenotes and caves of Quintana Roo and Yucatan.

###### Remarks.

*Antromysis cenotensis* was present in all the cenotes studied, except for Cenote Cervera. Álvarez et al. (2015) mentions that *A. cenotensis* occurs mostly above or occasionally below the halocline up to a depth of 16 m. In the present study, the species was only observed in freshwater habitats, in some cases as deep as the scope of the survey. Our findings prove this species as a common representative of the stygofauna of Yucatan, as it was found in more than 95% of the visited sites. *Antromysis cenotensis* is listed as “threatened” in the Mexican Red List of Threatened Species (NOM-059 SEMARNAT 2010).

#### 

**Order: Amphipoda**

**Family: Hadziidae**

##### Mayaweckelia troglomorpha

Taxon classificationAnimaliaAmphipodaHadziidae

Angyal, 2018

865140AA-36C8-566F-90F7-6148C7B6B288

Figure 2E

###### Material examined.

2 individuals; **Dzonbakal**, depth 26.3 and 26.5 m, cave, freshwater, 27 °C, San Antonio Mulix, Yucatan, Mexico; 14 May 2016; colls. R. Acosta, D. Angyal, J. Baduy & S. Reyes. 1 individual; **Cenote Kanun**, depth 24.3 m, cave, freshwater, 26 °C, Homun, Yucatan, Mexico; 4 June 2016; colls. R. Acosta, D. Angyal, J. Baduy, B. Magaña & S. Reyes. 1 individual; **Cenote Xaan**, depth 25.4 m, cave, freshwater, 27 °C, Homun, Yucatan, Mexico; 9 June 2016; colls. D. Angyal & E. Chávez Solís. 2 individuals; **Cenote Kankirixche**, depth 20.4 and 33.3 m, cavern and cave, freshwater, 27 °C, Homun, Yucatan, Mexico; 11 June 2016; colls. D. Angyal & E. Chávez Solís. 5 individuals; **Dzonotila**, depth 11.0-17.7 m, cavern, freshwater, 27 °C, Mucuyche, Yucatan, Mexico; 20 November 2017; colls. D. Angyal, E. Chávez Solís, S. Drs & B. Magaña. 2 individuals; **Cenote X’kokob**, depth 4.0-10.0 m, cavern, freshwater, 26 °C, Ekmul, Yucatan, Mexico; 17 December 2017; colls. D. Angyal, E. Chávez Solís, S. Drs & B. Magaña. 2 individuals; **Cenote Chihuo Hol**, depth 8.0-27.2 m, cavern, freshwater, 27 °C, Mucuyche, Yucatan, Mexico; 26 January 2018; colls. D. Angyal, S. Drs, L. Liévano, B. Magaña & N. Simoes. 1 individual; **Cenote Yax-Kis**, depth 8.0 m, cave, freshwater, 27 °C, Mucuyche, Yucatan, Mexico; 27 January 2018; colls. D. Angyal, S. Drs & L. Liévano.

###### Previous distribution.

Angyal et al. 2018. Type locality is Dzonbakal (Yucatan. Allotype female is from Cenote Kankirixche, paratypes are from Dzonbakal and cenotes Kanun, Xaan and Kankirixche (all in Yucatan).

###### Remarks.

At present, collected material is available from eight localities and a small *M. troglomorpha* population was also observed in Cenote San Elias. All the individuals were found in freshwater habitats, both in cave and cavern sections, where water temperature was between 26 and 27 °C. In cenote Kankirixche, some individuals were observed below 45 meters in depth. As a species recently described by our research group, one of the outcomes of present expeditions. As *M. troglomorpha* was found in approximately 30% of the visited sites, it does not appear to be a rare freshwater stygobiotic element in the Yucatan cenotes.

##### Mayaweckelia cenoticola

Taxon classificationAnimaliaAmphipodaHadziidae

Holsinger, 1977

4B48420A-484E-511C-BF87-5098C266B5DB

Figure 2F

###### Material examined.

1 individual; **Cenote Ayun-Nah**, depth 14.0 m, cave, freshwater, 27 °C, Cacalchen, Yucatan, Mexico; 22 May 2016; colls. D. Angyal, B. Magaña & E. Sosa Rodríguez. 1 individual; **Dzonotila**, depth 18.0 m, cavern, freshwater, 27 °C, Mucuyche, Yucatan, Mexico; 20 November 2017; colls. D. Angyal. E. Chávez Solís, S. Drs & B. Magaña. 1 individual; **Cenote Ixim Ha**, depth 4.7 m, cavern, freshwater, 25 °C, Tixkakal, Yucatan, Mexico; 25 November 2017; colls. D. Angyal, E. Chávez Solís, S. Drs, L. Liévano & E. Sosa. 3 individuals; **Cenote Bebelchen**, depth 0.5-7.3 m, cavern, freshwater, in water column and in roots at cavern entrance, 25 °C, Sanahcat, Yucatan, Mexico; 18 December 2017; colls. D. Angyal, S. Drs, L. Liévano & S. Reyes.

###### Previous distribution.

Holsinger 1977, 1990; Reddell 1981; Álvarez and Iliffe 2008, Álvarez et al. 2015, Angyal et al. 2018, Benítez et al. 2019.

Type locality is Cenote Xtacabiha (Yucatan). From Yucatan the species was also known from Cueva de Orizaba, Cenote Nohchen, Grutas de Tzab-Nah and Grutas de Santa Maria. From Quintana Roo there were records from Cenote Actun Ha (Carwash), Cenote de las Ruinas, Cenote de San Martin, Cenote de Santo Domingo, Cueva de Tancah, Odyssey, Bang and Tabano. From the state of Campeche, the species was known from the Volcán de los Murciélagos cave.

###### Remarks.

*Mayaweckelia cenoticola* proved to be rarer than *M. troglomorpha*, since it was recorded from only four cenotes. In Cenote Bebelchen we found some individuals in the roots of trees near the surface at the entrance region. Holsinger (1990) found that the species is associated mainly with freshwater habitats, with few populations occurring in weak brackish water. Individuals found in the Ox Bel Ha System (Quintana Roo) by Álvarez et al. (2015) and Benítez et al. (2019) also occurred in freshwater.

##### Tuluweckelia cernua

Taxon classificationAnimaliaAmphipodaHadziidae

Holsinger, 1990

0C127343-B2D9-51EA-A750-3B410F1F634D

Figure 2G

###### Material examined.

3 individuals; **Cenote San Juan**, depth 27.0-27.1 m, cave, freshwater, 27 °C, Homun, Yucatan, Mexico; 7 May 2016; colls. D. Angyal & E. Chávez Solís. 2 individuals; **Cenote Dzonbakal**, depth 29.0 m, cave, freshwater, 27 °C, San Antonio Mulix, Yucatan, Mexico; 22 May 2016; colls. D. Angyal, J. Baduy & B. Magaña. 10 individuals; **Cenote Tres Oches**, depth 15.8-22.9 m, cave, freshwater, 27 °C, Homun, Yucatan, Mexico; 5 June 2016; colls. D. Angyal & E. Chávez Solís. 3 individuals; **Cenote Xaan**, depth 22.7-26.6 m, cave, freshwater, 27 °C, Homun, Yucatan, Mexico; 9 June 2016; colls. D. Angyal & E. Chávez Solís. 3 individuals; **Cenote Kakuel**, depth 32.2-38 m, cave, freshwater, 27 °C, Mucuyche, Yucatan, Mexico; 10 June 2016; colls. D. Angyal & E. Chávez Solís. 3 individuals; **Cenote Kankirixche**, depth 20.4-49.6 m, cavern and cave, freshwater, 27 °C, Mucuyche, Yucatan, Mexico; 11 June 2016; colls. D. Angyal & E. Chávez Solís. 2 individuals; **Cenote Santito**, depth 5.3-6.0 m, cavern, freshwater, 27 °C, Kopoma, Yucatan, Mexico; 10 November 2017; colls. D. Angyal, S. Drs & L. Liévano. 1 individual; **Cenote X’baba**, depth 26.0 m, cavern, freshwater, 27 °C, Chochola, Yucatan, Mexico; 26 November 2017; colls. S. Drs, L. Liévano & E. Sosa. 1 individual; **Cenote Sabtun 1**, depth 25.0 m, cavern, above the halocline, 25 °C, Chunchumil, Yucatan, Mexico; 10 December 2017; colls. D. Angyal, S. Drs, E. Chávez Solís, Q. Hernández & S. Reyes. 1 individual; **Cenote Pixton**, depth 7.0 m, cavern, freshwater, 26 °C, Huhi, Yucatan, Mexico; 18 December 2017; colls. D. Angyal & L. Liévano. 3 individuals; **Cenote Yax-Kis**, depth 23.4-32.0 m, cave, freshwater, 27 °C, Mucuyche, Yucatan, Mexico; 27 January 2018; colls. D. Angyal, S. Drs & L. Liévano.

###### Previous distribution.

Holsinger 1990; Álvarez and Iliffe 2008; Álvarez et al. 2015; Angyal et al. 2018; Benítez et al. 2019.

Type locality is Cenote Calavera (Temple of Doom) in Quintana Roo. This species was known only from coastal caves of Quintana Roo: Mayan Blue, Actun Ha (Carwash), Mojara, Naharon (Cristal), Na’ach Wennen Ha, Bang, Muknal, Odyssey, and Tabano.

###### Remarks.

*Tuluweckelia cernua* was both the most frequent and abundant stygobiotic amphipod in the present study. Additional observations were from cenotes Yaal Utsil, El Virgen, and Dzalbay. In contrast with previous reports (e.g. Holsinger 1990), *T. cernua* always occurred in freshwater habitats. Individuals were collected between depths of 5-50 m. The species co-occurred with *M. troglomorpha* in five cenotes. These are the first distributional records of *T. cernua* for the state of Yucatan. Known localities of this species have almost tripled, increasing its distribution range into the Yucatan inland area.

#### 

**Order: Isopoda**

**Family: Cirolanidae**

##### Creaseriella anops

Taxon classificationAnimaliaIsopodaCirolanidae

(Creaser, 1936)

1245A348-F02A-5B3C-97E7-CEA84F5EE8FD

Figure 3A

###### Material examined.

3 individuals; **Cenote San Juan**, depth 20.0-28.0 m, cavern and cave, freshwater, 27 °C, Homun, Yucatan, Mexico; 7 May 2016; colls. D. Angyal & E. Chávez Solís. 1 individual; **Cenote Cervera**, depth 24.0 m, cave, below halocline, 26 °C, Yalsihom, Yucatan, Mexico; 8 May 2016; colls. D. Angyal & E. Chávez Solís. 2 individuals; **Cenote Tza Itza**, depth 12.5-13.5 m, cavern, freshwater, 27 °C, Tecoh, Yucatan, Mexico; 10 May 2016; colls. D. Angyal & E. Chávez Solís. 2 individuals; **Cenote Tres Oches**, depth 18.2-21.7 m, cave, freshwater, 27 °C, Homun, Yucatan, Mexico; 5 June 2016; colls. D. Angyal & E. Chávez Solís. 1 individual; **Cenote Kankirixche**, depth 3.0 m, cavern, freshwater, 27 °C, Mucuyche, Yucatan, Mexico; 11 June 2016; colls. D. Angyal & E. Chávez Solís. 1 individual; **Cenote Chihuo Hol**, depth 15.0 m, cavern, freshwater, 27 °C, Mucuyche, Yucatan, Mexico; 26 January 2018; colls. D. Angyal, S. Drs, L. Liévano, B. Magaña & N. Simoes.

###### Previous distribution.

Creaser 1936, 1938; Nicholas 1962; Reddell 1977, 1981; Holsinger 1990; Iliffe 1992, 1993; Fiers et al. 1996; Rocha et al. 1998; Botosaneanu & Iliffe 1999, 2002; Álvarez et al. 2005; Iliffe & Botosaenau 2006; Álvarez and Iliffe 2008; Sánchez-Rodríguez 2008; Ruiz-Cancino et al. 2013; Álvarez et al. 2015; Ortiz and Chazaro-Olvera 2015; Benítez et al. 2019.

Type locality is Cenote Sambula (Motul, Yucatan). Known from numerous caves and cenotes in Quintana Roo and Yucatan, and a well in Campeche.

###### Remarks.

The species was also observed in cenotes Yaal Utsil, Pol Box, X’kokob, Bebelchen, Kankal, San Elias, Dzonotila, Yax-Kis, Xaan and X’baba. *Creaseriella anops* was found both in cavern and cave sections, between 3 and 40 m deep. Our observations generally agree with the records of Iliffe and Botosanenau (2006) and Álvarez et al. (2015) as a freshwater species. However, as Benítez et al. (2019) reported, we also observed individuals around or below the halocline. *Creaseriella anops* is listed as “threatened” in the Mexican Red List of Threatened Species (NOM-059-SEMARNAT 2010).

##### Yucatalana robustispina

Taxon classificationAnimaliaIsopodaCirolanidae

Botosaneanu & Iliffe, 1999

C2143154-36B4-509E-A25E-70D02C104558

Figure 3B

###### Material examined.

1 individual; **Cenote Xaan**, depth 27.6 m, cave, freshwater, 27 °C, Homun, Yucatan, Mexico; 9 June 2016; colls. D. Angyal & E. Chávez Solís. 1 individual; **Cenote Kakuel**, depth 19.9 m, cavern, freshwater, 27 °C, Mucuyche, Yucatan, Mexico; 10 June 2016; colls. D. Angyal & E. Chávez Solís. 5 individuals; **Cenote Kankirixche**, depth 20-49.3 m, cavern and cave, freshwater, 27 °C, Mucuyche, Yucatan, Mexico; 11 June 2016; colls. D. Angyal & E. Chávez Solís. 3 individuals; **Cenote Kankirixche**, depth 10.0-27.0 m, cavern, freshwater, 27 °C, Mucuyche, Yucatan, Mexico; 26 January 2018; colls. D. Angyal, S. Drs, L. Liévano & B. Magaña. 1 individual; **Cenote Yaal Utsil**, depth 35.5 m, cave, freshwater, 27 °C, Mucuyche, Yucatan, Mexico; 3 November 2017; colls. D. Angyal, S. Drs & E. Chávez Solís. 1 individual; **Cenote Tza Itza**, depth 15.0 m, cavern, freshwater, 27 °C, Tecoh, Yucatan, Mexico; 3 November 2017; colls. D. Angyal, S. Drs & L. Liévano. 1 individual; **Cenote Pol Box**, depth 3.0 m, cavern, freshwater, 27 °C, Chochola, Yucatan, Mexico; 12 November 2017; colls. D. Angyal, S. Drs, L. Liévano & E. Sosa. 2 individuals; **Dzonotila**, depth 14.0 and 16.0 m, cavern, freshwater, 27 °C, Mucuyche, Yucatan, Mexico; 12 November 2017; colls. D. Angyal, S. Drs, E. Chávez Solís & B. Magaña. 1 individual; **Cenote X’baba**, depth 12.0 m, cave, freshwater, 25 °C, Chochola, Yucatan, Mexico; 12 November 2017; colls. S. Drs, L. Liévano & E. Sosa. 1 individual; **Cenote El Virgen**, depth 12.6 m, cavern, freshwater, 26 °C, Sotuta, Yucatan, Mexico; 20 December 2017; colls. L. Liévano & N. Simoes. 1 individual; **Cenote Chihuo Hol**, depth 20.6 m, cavern, freshwater, 27 °C, Mucuyche, Yucatan, Mexico; 26 January 2018; colls. D. Angyal, S. Drs, L. Liévano, B Magaña & N. Simoes. 3 individuals; **Cenote Yax Kis**, depth 12.0-33.0 m, cave, freshwater, 27 °C, Mucuyche, Yucatan, Mexico; 27 January 2018; colls. D. Angyal, S. Drs & L. Liévano.

###### Previous distribution.

Botosaneanu and Iliffe 1999, 2002, 2006; Álvarez and Iliffe 2008.

Type locality is Cenote Pabakal (Papakal), Yucatan. It was also found in cenotes Kankirixche, Kakuel, Chuih-Hol Dos, Xacha, and San Geronimo (all in Yucatan).

###### Remarks.

Individuals of *Y. robustispina* were collected in a third of all localities visited, where it occurred in freshwater between 3 and 49 m in depth. In eight cenotes *Y. robustispina* co-occurred with the isopod *C. anops*. Agreeing with our observations, previous records referred specimens caught in freshwater between 5-50 m in depth (Botosaneanu and Iliffe 1999, 2002, 2006). Known localities of this species have been doubled.

##### Cirolana yunca

Taxon classificationAnimaliaIsopodaCirolanidae

(Botosaneanu & Iliffe, 2000)

2CF22392-B621-577A-A5A4-5B01A2C2D402

Figure 3C

###### Material examined.

1 individual; **Cenote Tres Oches**, depth 22.4 m, cave, freshwater, 27 °C, Homun, Yucatan, Mexico; 5 June 2016; colls. D. Angyal & E. Chávez Solís. 1 individual; **Cenote X’baba**, depth 25.0 m, cave, freshwater, 25 °C, Chochola, Yucatan, Mexico; 26 November 2016; colls. S. Drs, L. Liévano & E. Sosa. 1 individual; **Cenote Chihuo Hol**, depth 19.0 m, cavern, freshwater, 27 °C, Mucuyche, Yucatan, Mexico; 26 January 2018; colls. D. Angyal, S. Drs, L. Liévano, B Magaña & N. Simoes. 1 individual.

###### Previous distribution.

Botosaneanu and Iliffe 2000, 2006; Álvarez and Iliffe 2008; Rocha-Ramírez et al. 2009.

Type locality is Cenote Sabak Ha (Yucatan). This species had only been collected from its type locality until our expeditions.

###### Remarks.

We here provide the first records after the original description, which was based on a single specimen collected at 60 m in depth near the halocline at a salinity of 1.4 g/l (Botosaneanu and Iliffe 2000, 2006). The three newly collected individuals were found in freshwater habitats, both in cavern and cave zones below 19 m in depth. The species was found in approximately 10% of the studied cenotes always as solitary individuals. Therefore, *C. yunca* seems to be a rare element of the Yucatan freshwater cenote ecosystems.

#### 

**Superorder: Eucarida**

**Order: Decapoda**

**Family: Atydae**

##### Typhlatya dzilamensis

Taxon classificationAnimaliaDecapodaAtydae

Álvarez, Iliffe & Villalobos, 2005

6F8784A2-37CF-5332-A31E-D1258B432CA5

Figure 3D

###### Material examined.

1 individual; **Cenote Cervera**, depth 27.4 m, cave, below halocline, 27 °C, Yalsihom, Yucatan, Mexico; 8 May 2016; colls. D. Angyal & E. Chávez Solís. 1 individual; **Cenote Sabtun 1**, depth 28 m, cavern, below halocline, 26 °C, Chunchumil, Yucatan, Mexico; 10 Dec 2017; colls. D. Angyal & E. Chávez.

###### Previous distribution.

Álvarez et al. 2005, 2015; Álvarez and Iliffe 2008; Benítez et al. 2019; Espinasa et al. 2019.

Type locality is Buya Uno, allotype was collected from Cenote Cervera and paratypes from Dzilamway, all cenotes in Dzilam de Bravo region (Yucatan north coast). This species was recently recorded at the Ox Bel Ha system south of Tulum (Benítez et al. 2019) and the Ponderosa system north of Tulum (Espinasa et al. 2019).

###### Remarks.

In accordance with previous records by Álvarez et al. (2005, 2015), our specimens were also collected in fully marine water. Recent observations of this species increase the expected distribution, suggesting an underground coastal and saline habitat that could extend from the south of Quintana Roo (Ox Bel Ha) to the west coast of Yucatan (Sabtun 1).

##### Typhlatya mitchelli

Taxon classificationAnimaliaDecapodaAtydae

Hobbs & Hobbs, 1976

83585C9F-260D-5F8E-A7B0-C29FFE6B2A74

Figure 3E

###### Material examined.

3 individuals; **Cenote San Juan**, depth 4.3-9.1 m, cave and cavern, freshwater, 27 °C, Homun, Yucatan, Mexico; 7 May 2016; colls. D. Angyal & E. Chávez Solís. 11 individuals; **Cenote Tza Itza**, depth 4.3-16.5 m, cave, freshwater, 27 °C, Tecoh, Yucatan, Mexico; 10 May 2016; colls. D. Angyal & E. Chávez Solís. 1 individual; **Cenote Dzonbakal**, depth 9.3 m, cavern, freshwater, 27 °C, San Antonio Mulix, Yucatan, Mexico; 14 May 2016; colls. R. Acosta, D. Angyal, J. Baduy & S. Reyes. 1 individual; 1 individual; **Cenote Dzonbakal**, depth 14 m, cavern, freshwater, 27 °C, San Antonio Mulix, Yucatan, Mexico; 29 May 2016; colls. D. Angyal, J. Baduy & B. Magaña. 5 individuals; **Cenote Kampepen**, depth 10.1 m, cavern, freshwater, 27 °C, Chinquila, Yucatan, Mexico; 17 May 2016; colls. D. Angyal & B. Magaña. 2 individuals; **Cenote Ayun-Nah**, depth 9 m, cave, freshwater, 27 °C, Cacalchen, Yucatan, Mexico; 22 May 2016; colls. D. Angyal, B. Magaña & E. Sosa Rodríguez. **Cenote Tres Oches**, depth 8.1-22 m, cave, freshwater, 27 °C, Homun, Yucatan, Mexico; 5 June 2016; colls. D. Angyal & E. Chávez Solís. 7 individuals; **Cenote Kakuel**, depth 5-25.8 m, cave and cavern, freshwater, 27 °C, Mucuyche, Yucatan, Mexico; 10 June 2016; colls. D. Angyal & E. Chávez Solís. 1 individual; **Cenote Kankirixche**, depth 30.2 m, cavern, freshwater, 27 °C, Mucuyche, Yucatan, Mexico; 10 December 2016; colls. D. Angyal & E. Chávez Solís. 2 individuals; **Cenote Sabtun 1**, depth 24.0 and 25.0 m, cavern, above the halocline, 25 °C, Chunchumil, Yucatan, Mexico; 10 December 2017; colls. D. Angyal, E. Chávez Solís, S. Drs, Q. Hernández & S. Reyes. 1 individual; **Cenote Bebelchen**, depth 34.0 m, cavern, freshwater, 25 °C, Sanahcat, Yucatan, Mexico; 18 December 2017; colls. D. Angyal, S. Drs, L. Liévano & S. Reyes. 1 individual; **Cenote El Virgen**, depth 19.9 m, cavern, freshwater, 26 °C, Sotuta, Yucatan, Mexico; 20 December 2017; colls. L. Liévano & N. Simoes. 1 individual; **Cenote Chihuo Hol**, depth 26.0 m, cavern, freshwater, 27 °C, Mucuyche, Yucatan, Mexico; 26 January 2018; colls. D. Angyal, S. Drs, B. Magaña, L. Liévano & N. Simoes.

###### Previous distribution.

Hobbs and Hobbs 1976; Hobbs et al. 1977; Hobbs 1979; Reddell 1977, 1981; Iliffe 1992; Rocha et al. 1998; Webb 2003; Botello and Álvarez 2013; Benítez 2014; Álvarez et al. 2015; Chávez Solís 2015; Benítez et al. 2019.

Type locality is Cenote Kabahchen (Yucatan). The species occurs in numerous caves and cenotes throughout the peninsula in Quintana Roo and Yucatan.

###### Remarks.

Our findings corroborate that *T. mitchelli* is a widespread common crustacean in the freshwater cenotes of Yucatan. This species was caught from the shallow zones to 34 m in depth, indicating a wide vertical range as well as a wide geographical range. The species was also observed (but not collected) in cenotes Yaal Utsil, Santito, Pol Box, Kankal, San Elias, Dzonotila, X’baba, X’kokob, Pixton, Dzalbay, and Yax-Kis. *Typhlatya mitchelli* is listed as “least concern” in the IUCN Red List (De Grave et al. 2013a) and as “threatened” in the Mexican Red List of Threatened Species (NOM-059-SEMARNAT 2010).

##### Typhlatya pearsei

Taxon classificationAnimaliaDecapodaAtydae

Creaser, 1936

8C11C12E-B818-5976-8AED-E5840A562141

Figure 3F

###### Material examined.

1 individual; **Cenote Tres Oches**, depth 21.6 m, cave, freshwater, 27 °C, Homun, Yucatan, Mexico; 6 June 2016; colls. D. Angyal & E. Chávez Solís. 2 individuals; **Cenote Xaan**, depth 25.8 and 26.1 m, cave, freshwater, 27 °C, Homun, Yucatan, Mexico; 9 June 2016; colls. D. Angyal & E. Chávez Solís. 1 individual; **Cenote Kankirixche**, depth 3 m, cavern, freshwater, 27 °C, Mucuyche, Yucatan, Mexico; 11 June 2016; colls. D. Angyal & E. Chávez Solís. **Cenote Nohmozon**, depth 12.2 m, cavern, freshwater, 25 °C, Pixyah, Tecoh, Yucatan, Mexico; 11 March 2016; colls. E. Chávez Solís.

###### Previous distribution.

Creaser 1936; Nicholas 1962; Hobbs et al. 1977; Holthuis 1977; Hobbs 1979; Reddell 1977, 1981; Pérez-Aranda 1983a; Holsinger 1990; Iliffe 1992; Webb 2003; Hunter et al. 2007; Yager and Madden 2010; Botello and Álvarez 2013; Mejía-Ortíz et al. 2013; Benítez 2014; Pakes et al. 2014; Álvarez et al. 2015; Chávez Solís 2015; Benítez et al. 2019.

Type locality is ‘Balam Canche Cave’ (Grutas de Balankanche, Yucatan). The species is widely distributed within the northern part of the Yucatan Peninsula; it occurs in Quintana Roo, Yucatan, and Campeche.

###### Remarks.

Despite previous studies stating that *T. pearsei* has the largest of *Typhlatya*’s distribution range in the Yucatan Peninsula (Álvarez et al. 2015), we only collected individuals in a few localities, where it occurred in freshwater, both near the surface in open cenote pools and in deeper cave passages up to 26 m in depth. This species is listed as “least concern” in the IUCN Red List (De Grave et al. 2013b) and as “threatened” in the Mexican Red List of Threatened Species (NOM-059-SEMARNAT 2010).

#### 

**Family: Palaemonidae**

##### Creaseria morleyi

Taxon classificationAnimaliaDecapodaPalaemonidae

(Creaser, 1936)

F113F0EC-7B78-5421-A327-142E8C65FB30

Figure 3G

###### Material examined.

2 individuals; **Cenote Tza Itza**, depth 15.4 m, cavern, freshwater, 27 °C, Tecoh, Yucatan, Mexico; 10 May 2016; colls. D. Angyal & E. Chávez Solís. 2 individuals; **Cenote Kampepen**, depth 6-9.5 m, cavern, freshwater, 27 °C, Chinquila, Yucatan, Mexico; 17 May 2016; colls. D. Angyal & B. Magaña. 2 individuals; **Cenote Kakuel**, depth 3 and 13.9 m, cavern, freshwater, 27 °C, Mucuyche, Yucatan, Mexico; 10 June 2016; colls. D. Angyal & E. Chávez Solís. 1 individual; **Cenote Kankirixche**, depth 3.6 m, cavern, freshwater, 27 °C, Mucuyche, Yucatan, Mexico; 11 June 2016; colls. D. Angyal & E. Chávez Solís. 1 individual; **Cenote Santito**, depth 4.0 m, cavern, freshwater, 27 °C, Kopoma, Yucatan, Mexico; 10 November 2017; colls. D. Angyal, S. Drs & L. Liévano. 1 individual; **Cenote Kankal**, depth 0.3 m, cavern, freshwater, 25 °C, Homun, Yucatan, Mexico; 18 November 2017; colls. D. Angyal, S. Drs, E. Chávez Solís & L. Liévano. 1 individual; **Cenote Bebelchen**, depth 30.0 m, cavern, freshwater, 25 °C, Sanahcat, Yucatan, Mexico; 18 December 2017; colls. D. Angyal, L. Liévano & S. Reyes. 1 individual; **Cenote El Virgen**, depth 25.0 m, cavern, freshwater, 26 °C, Sotuta, Yucatan, Mexico; 20 December 2017; colls. L. Liévano & N. Simoes. 1 individual; **Cenote Dzalbay**, depth 4.3 m, cavern, freshwater, 23 °C, Sotuta, Yucatan, Mexico; 20 December 2017; colls. D. Angyal & L. Liévano.

###### Previous distribution.

Creaser 1938; Hobbs and Hobbs 1976; Holthuis 1977; Hobbs et al. 1977; Reddell 1977, 1981; Hobbs 1979; Pérez-Aranda 1983b; Iliffe 1992; Botello and Álvarez 2006; Botello and Álvarez 2010; Benítez 2014; Álvarez et al. 2015; Chávez Solís 2015; Chávez Solís et al. 2017; Benítez et al. 2019.

Type locality is San Isidro Cave (Yucatan). Widely distributed in cenotes and caves of Yucatan, Campeche, and Quintana Roo.

###### Remarks.

Reddell (1981) mentions the species as an “ever-present element of fauna of pools and lakes in caves in the Yucatan Peninsula”. In addition to the above listed localities, we also observed the species in cenotes Yaal Utsil, Pol Box, San Elias, Dzonotila, Flor de Liz, X’baba, Chihuo Hol, and Yax-Kis. Specimens were recorded in both cave and cavern sections, up to 38 m in depth. Benítez et al. (2019) also found individuals around and below the halocline in cenotes belonging to the Ox Bel Ha system. *Creaseria morleyi* is listed as “threatened” in the Mexican Red List of Threatened Species (NOM-059-SEMARNAT 2010) and as “least concern” in the IUCN Red List (De Grave et al. 2013c).

## Discussion

While there are more than 3,000 registered cenotes in the state of Yucatan (SDS Yucatan census), less than five percent have been zoologically investigated. Results herein confirm that the region deserves more attention and that the geographical, bathymetric, and fresh/salt water distribution of stygobiotic species is far from being fully understood. In order to contribute to the management of the vulnerable cenote ecosystems and their highly specialized endemic stygofauna, collecting as much information as possible about the biology of Yucatan aquifers would be paramount. This data should include reports on the species’ distribution, density and rarity, taxonomy, ecology, as well as characteristics of their habitats related to their biology, such as the amount of epigean originated organic sources or the degree of anthropogenic pollution in cenotes.

Prior to this study, the amphipod *T. cernua* was only known from Quintana Roo, mostly associated with saltwater habitats in anchialine cenotes near the northeastern coastline of the Peninsula (Holsinger 1990; Rocha et al. 1998; Álvarez and Iliffe 2008; Álvarez et al. 2015). Contrary to previous findings, all individuals were found in freshwater habitats during our study (Angyal et al. 2018). Rocha et al. (1998) and Pesce and Iliffe (2002) mentioned observation records of ’thermosbaenaceans’ from cenotes Yuncu, Mucuyche, Pabakal (Papakal), and Grutas de Tzab-Nah (all in Yucatan). However, these individuals had never been identified at the species level and it seems no voucher information of the potentially collected specimens is available. The present study confirms first records for *T. cernua* and *T. unidens* in the state of Yucatan. Together with the amphipod *M. troglomorpha*, which was discovered and described within the frame of herein presented expeditions (Angyal et al. 2018) and the new cave isopod *Curassanthura yucatanensis* Álvarez, Benítez, Iliffe & Villalobos, 2019 (Álvarez et al. 2019), the list of stygobiotic crustaceans recorded for the state of Yucatan raised from 22 (in 2016) to 26. In addition, the cirolanid isopod *C. yunca* was only known from its type locality, but we now provide distribution data for this species in three other localities. Our results show that the stygiomysid S. cf. holthuisi has historically been unrecognized, unsampled or ignored. This specific contribution proves that inland cenotes have been understudied and distribution patterns of stygofauna are still unknown. Due to the previously lacking zoological information for the vast majority of the cenotes investigated in our study, most of the distribution records presented here are new.

A closer morphological and molecular analysis of the *Typhlatya* species in Yucatan is recommended in order to distinguish cryptic species that may be causing confounding biodiversity and ecological patterns in the Yucatan Peninsula.

Among the 14 crustacean species listed, prior to this study, cytochrome c oxidase subunit I sequences were publicly available only for the decapods *T. mitchelli*, *T. pearsei*, *T. dzilamensis*, and *C. morleyi*. The currently published COI barcode gene fragments can aid future molecular research on the peracarid fauna of Yucatan’s cenote ecosystems by facilitating their identification, as well as in the recognition of cryptic species.

The mysid *A. cenotensis*, the atyid shrimps *T. mitchelli* and *T. pearsei* and the palaemonid shrimp *C. morleyi* are listed in the Mexican and IUCN red lists of threatened species (SEMARNAT 2010; De Grave et al. 2013a, b, c). These species are present in most cenotes throughout the Yucatan Peninsula and can be considered a selected group of species whose protection will act as an umbrella in protecting other less common ones. On the other hand, there are rare species with an extremely narrow distribution range, which are not yet under legal protection. This makes these species even more vulnerable to urbanization and environment deterioration. Therefore, we suggest the inclusion of narrow endemic species into the national and international protection lists, such as the isopod *C. yunca* or the atyid shrimp *T. dzilamensis*.

The number of new records provided in this work shows a historic lack of biodiversity surveys in underwater caves of inland cenotes of the state of Yucatan. Most of the biodiversity and its distribution patterns are currently biased towards large populations, easily accessible sites, and touristic attractions. Our efforts yield a greater understanding of the distribution patterns of stygofauna in Yucatan cenotes.

## Supplementary Material

XML Treatment for Tulumella unidens

XML Treatment for Stygiomysis cokei

XML Treatment for Stygiomysis cf. holthuisi

XML Treatment for Antromysis cenotensis

XML Treatment for Mayaweckelia troglomorpha

XML Treatment for Mayaweckelia cenoticola

XML Treatment for Tuluweckelia cernua

XML Treatment for Creaseriella anops

XML Treatment for Yucatalana robustispina

XML Treatment for Cirolana yunca

XML Treatment for Typhlatya dzilamensis

XML Treatment for Typhlatya mitchelli

XML Treatment for Typhlatya pearsei

XML Treatment for Creaseria morleyi
